# Machine learning for prediction of childhood mental health problems in social care

**DOI:** 10.1192/bjo.2025.32

**Published:** 2025-04-11

**Authors:** Ryan Crowley, Katherine Parkin, Emma Rocheteau, Efthalia Massou, Yasmin Friedmann, Ann John, Rachel Sippy, Pietro Liò, Anna Moore

**Affiliations:** New York University Grossman School of Medicine, New York, US; Department of Public Health and Primary Care, University of Cambridge, Cambridge, UK; Department of Psychiatry, University of Cambridge, Cambridge, UK; Cambridge Public Health, University of Cambridge, Cambridge, UK; Department of Computer Science, University of Cambridge, Cambridge, UK; Neath Port Talbot County Borough Council, Port Talbot, UK; Population Psychiatry, Suicide and Informatics, Swansea University Medical School, Swansea, UK; Anna Freud, London, UK; Cambridgeshire and Peterborough NHS Foundation Trust, Cambridge, UK

**Keywords:** Mental health services, medical technology, community mental health teams, machine learning methods, precision medicine

## Abstract

**Background:**

Rates of childhood mental health problems are increasing in the UK. Early identification of childhood mental health problems is challenging but critical to children’s future psychosocial development. This is particularly important for children with social care contact because earlier identification can facilitate earlier intervention. Clinical prediction tools could improve these early intervention efforts.

**Aims:**

Characterise a novel cohort consisting of children in social care and develop effective machine learning models for prediction of childhood mental health problems.

**Method:**

We used linked, de-identified data from the Secure Anonymised Information Linkage Databank to create a cohort of 26 820 children in Wales, UK, receiving social care services. Integrating health, social care and education data, we developed several machine learning models aimed at predicting childhood mental health problems. We assessed the performance, interpretability and fairness of these models.

**Results:**

Risk factors strongly associated with childhood mental health problems included age, substance misuse and being a looked after child. The best-performing model, a gradient boosting classifier, achieved an area under the receiver operating characteristic curve of 0.75 (95% CI 0.73–0.78). Assessments of algorithmic fairness showed potential biases within these models.

**Conclusions:**

Machine learning performance on this prediction task was promising. Predictive performance in social care settings can be bolstered by linking diverse routinely collected data-sets, making available a range of heterogenous risk factors relating to clinical, social and environmental exposures.

## Childhood mental health problems

The burden of childhood mental health problems is increasing in the UK, with a recent report placing the prevalence at approximately 16%.^1^ This increase may stem from a confluence of factors including the COVID-19 pandemic, widening income inequality, social media usage and increased pressure within school settings.^2^ Children in social care settings have a greater risk of poor mental health outcomes, which may be explained by more frequent exposure to adverse childhood experiences (ACEs) and barriers to accessing care.^3^ Identifying childhood mental health problems is difficult, particularly for non-specialists, because early symptoms of a disorder can be challenging to disentangle from normal development, children experience different symptoms as they age and they may struggle to explain their feelings and behaviours.^4^ Mental health problem identification for children with social care contact can be particularly difficult because ACEs can negatively impact development, and the care systems normally responsible for identifying problems in children (e.g. carers, general practitioners and schools) are inconsistent and disrupted. Estimates on the rates of mental health problems in children in social care settings vary, with some figures ranging from 19 to 38%.^5,6^ Despite the importance of early detection of mental health problems to facilitate provision of appropriate support, children with social care contact struggle to access assessment and subsequent treatment. This perpetuates difficulties as children’s early experience with psychopathology can lead to negative outcomes that affect them throughout adolescence and adulthood.^7,8^ It is therefore imperative to develop alternative solutions to support the early identification of problems for this vulnerable group.

## Clinical prediction tools in psychiatry

Despite the increasing burden of mental health problems on healthcare systems, growth in the number of mental health professionals is significantly outpaced by those afflicted.^9^ Clinical prediction tools can potentially improve outcomes and reduce resource burdens by identifying mental health problems early and guiding individuals toward appropriate support. There is a growing body of literature describing predictive models for mental health problems such as depression, suicide and anxiety disorders.^10-12^ Nevertheless, there is an expanding need for effective tools for the prediction of mental health problems in children, and none have yet been translated into clinical use.^13^ Discrepancies between the vast potential for machine learning applications and corresponding lack of improvement in patient outcomes has been dubbed the ‘artificial intelligence chasm’. Low-quality evaluations of model performance are common and an important cause of the chasm; evaluations are often conducted via internal validation using methods that may overestimate performance.^14^ These internal validation methods are most problematic when performed without proper safeguards to ensure accurate model performance estimation. These issues are often magnified within psychiatry, where models typically suffer from low generalisability as assessments are predominantly conducted in homogeneous populations in affluent countries, data-sets are typically smaller and external validations of model performance are uncommon.^15–17^ Using population-based, representative data-sets based on data that is routinely collected can mitigate some of these limitations.

## Secure Anonymised Information Linkage Databank

The Secure Anonymised Information Linkage (SAIL) Databank is a national data safe haven, providing approved researchers with linkable de-identified health, social care and education data-sets relating to the Welsh population.^18^ The Adolescent Mental Health Data Platform (ADP) contains data relating to children and young people, and includes routinely collected data on demographics, education (e.g. attendance and attainment), health (e.g. out-patient care) and social care contact (e.g. child protection records). These data-sets contain various risk factors pertinent to mental health problems that can be used for model building. For social care, Children In Need Wales (CINW) was succeeded by Children Receiving Care and Support (CRCS) following the enactment of the Social Services and Well-being (Wales) Act in April 2016. Both data-sets utilise ‘need for care and support’ as the all-encompassing indication for inclusion of children within the data-set, and employ annual census collection methods that differ slightly in implementation. See Lee et al for details of these data-sets.^19^

## Study aims

We aimed to develop prototype machine learning models for the prediction of mental health problems in children under social care services, using the SAIL Databank. Since artificial intelligence algorithms can reinforce historical patterns of systemic bias,^20^ we took an approach that integrates clinician perspectives, focuses on model interpretability and assesses algorithmic fairness.

## Method

### Data

This study was reported according to the TRIPOD+AI framework.^21^ The checklist can be found in Supplementary Table 1 (available at https://doi.org/10.1192/bjo.2025.32). With support from the ADP, we linked 18 data-sets from the SAIL Databank (Supplementary Table 2). This linking process utilised demographic information and local identifiers to connect individuals to a unique anonymous linkage field identifier. Individuals were eligible for inclusion if they were aged 10–17 years within the years 2013–2020 and had social care contact at any time (i.e. appeared in either the CINW or CRCS data-sets). Individuals were excluded if they were under 10 years old (because the social care data-sets only categorise children 10 years or older as having mental health problems), could not be linked to the other data-sets or information on their mental health status was not available in the social care data-sets. All retrospective data relating to these young people were included for analysis. Nested cross-validation was performed, a technique involving two levels of cross-validation that allows for both optimisation of model hyperparameters and estimation of model performance. Ten-fold cross-validation was performed for the outer loop and five-fold cross-validation was performed for the inner loop. A fixed random seed was used for reproducibility.

### Mental health outcomes

Data collected on mental health events by the CINW/CRCS censuses were utilised for measurement of the outcome. As defined by CINW/CRCS, a child had a mental health problem if they were 10 years or older and met any of the following criteria: had been diagnosed by a medical practitioner, had received child and adolescent mental health services (CAMHS) or were on a waiting list for CAMHS. Mental health problems included depression, anxiety, eating disorders, self-harm and other disorders, but excluded substance misuse, autism spectrum disorders and other intellectual disabilities unless accompanied by mental health problems.

### Diagnosis/intervention codes

Diagnostic codes within the SAIL Databank follow the ICD-10 format.^22^ Intervention codes within the SAIL Databank follow the format of the Office of Population Censuses and Surveys Classification of Surgical Operations and Procedures, Version Four. This classification contains hierarchical codes for interventions and procedures undertaken by the National Health Service. We removed codes beginning with ‘F’ within the ICD-10, which relate to psychiatric or neurological disorders, or both, as this was our outcome of interest. We also removed other codes directly related to our outcomes such as those beginning with ‘X6’, which is intentional self-harm. To maintain the hierarchical structure of diagnoses and interventions, we assigned different features to each class level (e.g. ‘G1’, ‘G12’, ‘G12.1’) and used one-hot encodings with each unique encoding referring to presence versus absence of a particular diagnosis. To maintain a manageable level of sparsity while retaining the largest amount of useful clinical information, only diagnosis and intervention codes with a prevalence within the cohort of 2% or greater were retained. If a diagnosis did not meet this threshold, it was still included via all parent classes that qualify (e.g. G12.1 had a prevalence below the threshold of 0.4%, but its parent class G12 had a frequency of 6%, so it was retained).

### Risk factors

We utilised a framework previously developed by the team through a rapid literature review and Delphi process. The framework contains 287 risk factors, grouped into seven domains: social and environmental, behavioural, education and employment, biomarkers, physical health, psychological and mental health, and patterns of service use (Supplementary Table 3a–3h). An eighth domain combines the risk factors from these domains that are particularly relevant for underserved populations, and is intended to reduce bias in model development through the inclusion of salient risk factors for underserved populations. A mapping exercise between the Delphi risk factor framework to SAIL Databank metadata established that 110 of the 287 (38.33%) were measurable. Of these, 41 met the missing values criteria of having data for at least 20% of the cohort and were included in the final model (Supplementary Table 4). Some of these risk factors (e.g. ethnicity) had multiple categories, thus there were more categorical features in the model than original risk factors. The final risk factors included correspond to six continuous features and 69 categorical features in the model. Exploration of comorbid diagnoses and chronic medical conditions from the Patient Episode Database for Wales yielded 2643 unique diagnostic codes and 1185 unique intervention codes. A total of 83.04% of children had at least one diagnosis listed and 55.08% had at least one intervention listed. Sixty unique diagnoses and 23 unique interventions met the 2% prevalence cut-off and were included within the model as features. Together, these provided 158 features that were used for modelling.

Risk factors with values at multiple time points were converted into binary variables indicating whether the individual had ever been exposed to the risk factor. For children with a mental health problem, risk factor data were only included if it occurred temporally before the first positive recorded instance of a mental health problem. For children without a mental health problem, all information was included for prediction up to the final date that they had social care data. Given this is a real-world clinical data-set, there were substantial missing data. If there were missing data regarding a risk factor, individuals were categorised as ‘unknown’ for that risk factor, and this was included as a feature for the models. This approach was chosen because it provides full flexibility to the models by allowing them to weigh the importance of missing data. Advantages and limitations of this approach are explored in the ‘Discussion’ section.

To reduce multicollinearity and subsequently improve interpretability, categorical risk factors were represented as one-hot encodings. Continuous variables were standardised using sample means and standard deviations, with absolute cut-offs applied at ±4 s.d. from the mean, to remove errors and extreme anomalies. If a value for a continuous variable was missing, it was set to the mean of that variable. If a continuous variable had some missing data, an additional binary variable was created to indicate missing data. Including ethnicity in a predictive model that supports decision-making regarding care access has potential equity ramifications. However, given our exploratory focus, we retained ethnicity data to gain insight into how to create equitable classifiers.

### Modelling decisions

Many machine learning methods applied to clinical data-sets have shown success by utilising recurrent neural network model structures to model time-series data.^23-25^ However, most of our data-sets were derived from annual censuses that were not sufficiently granular to merit a time-series analysis. Prior research has demonstrated the labels of a psychiatry diagnosis are insufficient for modelling since psychiatric diseases are often heterogeneous, multifactorial and highly comorbid.^15,16^ Further, transdiagnostic interventions relating to prevention and treatment of childhood mental health problems demonstrate efficacy regardless of the underlying pathology.^26^ Thus, we framed the prediction task as a binary classification problem (i.e. the presence or absence of a mental health problem). This fits well with the clinical problem – we aim to build a tool for use by social workers to systematically identify which children might need referral to CAMHS, rather than replace the need for assessment and management within the specialist mental health setting.

Within the cohort, a minority of the children had a mental health problem, so a model could achieve high accuracy by classifying all individuals as healthy. Since such a model would not have clinical utility, loss functions were adjusted to apply greater emphasis (weight) to the correct classification of children with a mental health problem. The standard formulas for weighting to obtain balanced class performances are shown in Equation 1 for those with a mental health problem, and Equation 2 for those without. No additional calibration was performed.
(1)

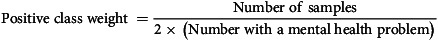



(2)

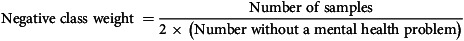




Model outputs for all models are probabilities. Thresholds were identified using the above equations to ensure adequate recall of the minority class. Because of its interpretability, logistic regression was used as the baseline model. Other standard models implemented included support vector machine (SVM) with radial basis function kernel, random forest, multilayer perceptron (MLP) and gradient boosting classifiers. Part of our study was to explore if machine learning approaches improved performance relative to logistic regression. These additional models are more complex and have associated model ‘hyperparameters’ (e.g. size of the model) whose values are fixed before the model is trained. The models were created with the Python package Scikit-Learn Version 1.6 for Windows.^27^ The class weighting formulas were not applied to the gradient boosting models and MLP models as their formulations in Scikit-Learn do not allow for class balancing. No feature selection was performed for the models. The hyperparameter search space and values of the optimised hyperparameters are shown in Supplementary Table 5.

### Performance metrics

Because of our unbalanced data-set, we used area under the receiver operating characteristic curve (AUROC) as the primary evaluation metric. AUROC can be interpreted as the probability that a classifier will rank a randomly chosen positive instance higher than that of a randomly chosen negative instance.^28^ We also reported area under the precision–recall curve (AUPRC) as a supplementary evaluation metric.

### Fairness metrics

An important aspect of evaluation is to explore bias or differing performance between subgroups. We utilised common fairness metrics (equalised odds and predictive parity) to gain insights into model performance for populations that differed with regards to two salient characteristics: gender and ethnicity. Equalised odds parity is satisfied when the true positive rate (TPR), also known as sensitivity, and the true negative rate (TNR), also known as specificity, are equivalent for the groups of interest. Predictive parity, in contrast, is satisfied when the positive predictive value (PPV) and negative predictive value (NPV) are equivalent for the groups of interest.^29^

### Ethics

Our application to obtain access to the SAIL Databank was reviewed and approved by their internal and external Information Governance Review Panel. Since all data-sets were anonymised and there was statistical disclosure control for outputs (e.g. reported results must include a minimum of five individuals), there was no legal requirement for the obtainment of individual consent.

## Results

### Cohort description

The baseline cohort included a sample of 1 113 776 children, of which 46 744 (4.20%) had social care contact. Individuals were excluded if they were under 10 years old (*n* = 17 992; 38.49%), could not be linked to the other data-sets (*n* = 1753; 3.75%) or data regarding their mental health status were not available (*n* = 149; 0.32%). This reduced the final cohort size to 26 820 individuals (57.38% of those with social care contact). Demographic information is shown in Table 1. Chi-squared tests were performed to identify if differences between the two groups were statistically significant.

**Table 1:** Cohort demographics

Variable	Total data-set, *n* (%)	Children with a mental health problem, *n* (%)^a^	Children without a mental health problem, *n* (%)^b^	*χ* ^2^-value	*P*-value
Sample size	26 820 (100)	5303 (19.77)	21 517 (80.23)		
Age, years				300	<0.00001
10–12	8845 (32.98)	1374 (25.91)	7471 (34.72)	150	<0.00001
13–15	9801 (36.54)	2441 (46.03)	7360 (34.21)	257	<0.00001
16–18	7868 (29.34)	1466 (27.64)	6402 (29.75)	9.12	<0.01
19–21	306 (1.14)	22 (0.41)	284 (1.32)	30.9	<0.00001
Gender				34.5	<0.00001
Girls	14 283 (53.26)	2633 (49.65)	11 650 (54.14)		
Boys	12 537 (46.74)	2670 (50.35)	9867 (45.86)		
Ethnicity				39.0	<0.00001
Asian	485 (1.81)	56 (1.06)	429 (1.99)	21.1	<0.00001
Black	314 (1.17)	45 (0.85)	269 (1.25)	5.93	<0.05
Mixed	696 (2.60)	139 (2.62)	557 (2.59)	0.018	0.894
White	24 010 (89.52)	4840 (91.27)	19 170 (89.09)	21.5	<0.00001
Other	263 (0.98)	36 (0.68)	227 (1.05)	6.20	<0.05
Not obtained	886 (3.30)	163 (3.07)	723 (3.36)	1.09	0.296
Refused to say	166 (0.62)	24 (0.45)	142 (0.66)	2.97	0.085
Free school meal status				8.59	<0.05
Eligible	20 253 (75.51)	3922 (73.96)	16 331 (75.90)	7.83	<0.01
Not eligible	6460 (24.09)	1360 (25.65)	5100 (23.70)	8.79	<0.01
Unknown	107 (0.40)	21 (0.40)	86 (0.40)	0.0009	0.977

a. Children with an identified mental health problem.

b. Children without an identified mental health problem.

The mean age among children who experienced a mental health problem was 14.5 years (s.d. 2.15 years). Children aged 13–15 years were most commonly found to have mental health problems. There was a higher prevalence of mental health problems in boys (21.30%) compared with girls (18.43%). Given the class imbalance, the weight given by Equation 1 was 2.53 and the weight given by Equation 2 was 0.62, corresponding to upweighting the class of individuals with a mental health problem by 4.06. The most common ethnicity within the data-set was White (89.52%). The demographics of this data-set are similar to the overall demographics of Wales recorded in the 2011 Census.^30^

### Model performance

The performance of the models is shown in Table 2. Confidence intervals were calculated with sample means from the ten outer cross-validation runs.

**Table 2:** Model performance on ten-fold cross-validation with 95% confidence intervals

Model	AUROC cross-validation (95% CI)	AUPRC cross-validation (95% CI)
Logistic regression	0.72 (0.69–0.74)	0.41 (0.38–0.45)
Support vector machine	0.75 (0.73–0.78)	0.46 (0.43–0.49)
Random forest	0.73 (0.70–0.76)	0.43 (0.39–0.47)
Gradient boosting classifier	0.75 (0.72–0.79)	0.47 (0.43–0.51)
Multilayer perceptron	0.72 (0.68–0.75)	0.44 (0.40–0.48)

AUROC, area under the receiver operating characteristic curve; AUPRC, area under the precision–recall curve.

The best-performing model was the gradient boosting classifier, which achieved an AUROC of 0.75 and an AUPRC of 0.47; the next best-performing model was the SVM, with an AUROC of 0.75 and an AUPRC of 0.46. There was a high degree of concordance between the AUROC and AUPRC performance. The worst-performing models were the logistic regression model and the MLP model.

### Model interpretability

To understand the risk factors most closely associated with adverse mental health outcomes, we performed an interpretability analysis with the best-performing model, the gradient boosting classifier.

**Fig. 1:**
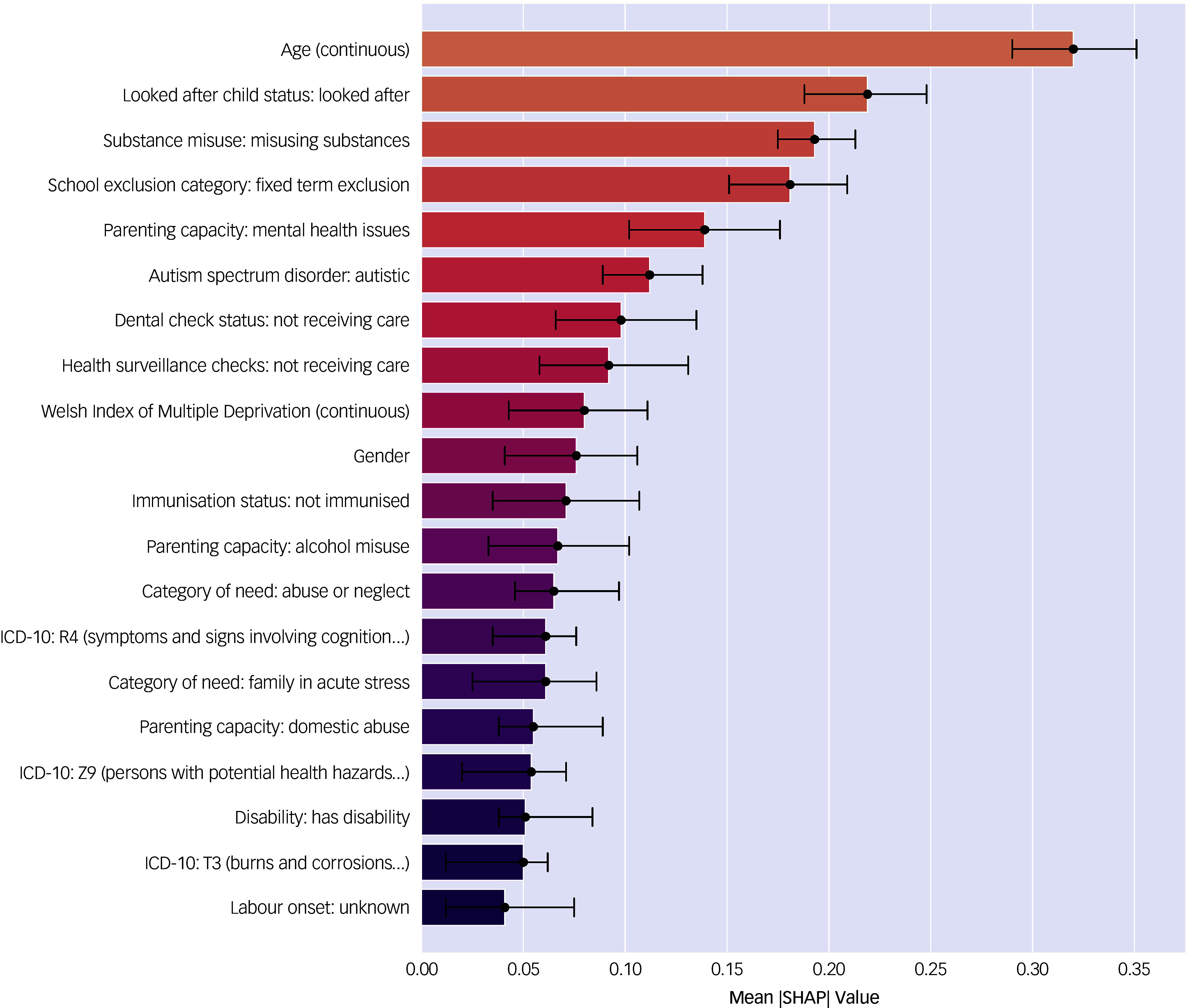
Mean absolute SHapley Additive exPlanations (SHAP) values for best-performing gradient boosting classifier.

Shapley values, which are a method for local interpretation originally developed for game theory, can help provide estimates of variable importance for non-linear machine learning methods.^31^ Calculating the mean absolute SHapley Additive exPlanations (SHAP) values across the entire test data-set can provide global interpretability regarding the relative importance of different features. SHAP values were calculated with the SHAP python package TreeExplainer method.^32^ The 20 most important features based on SHAP values are shown below in Fig. 1, using an 80–20 train-test split. Higher SHAP values indicate increased feature importance. Confidence intervals were derived by bootstrapping the training data-set 500 times and re-running SHAP calculations.

**Fig. 2:**
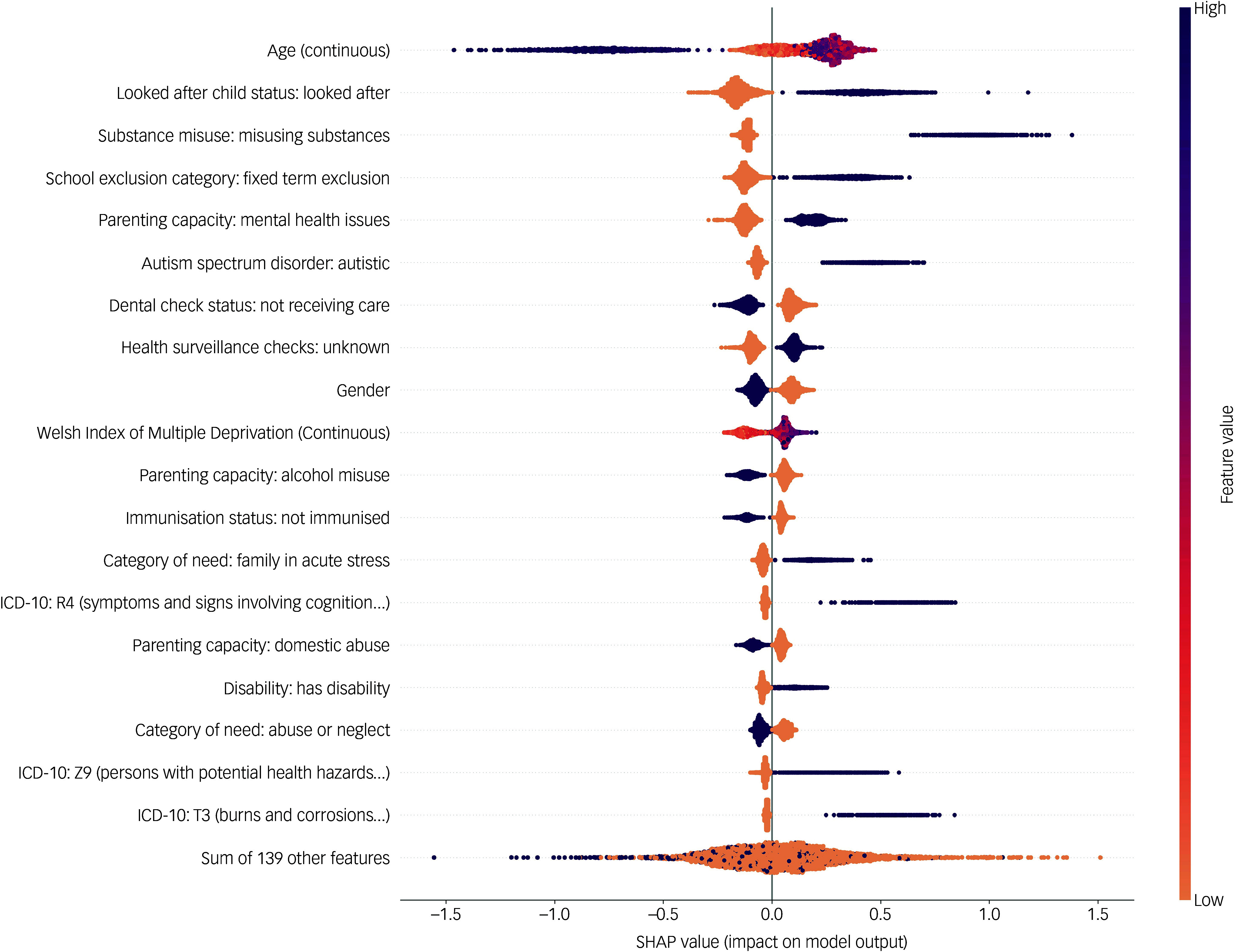
SHapley Additive exPlanations (SHAP) beeswarm plot for best-performing gradient boosting classifier.

The five risk factors with the largest mean SHAP values were age, looked after child status: looked after, substance misuse: misusing substances, school exclusion category: fixed term exclusion and parenting capacity: mental health issues. The complete list of 158 mean absolute SHAP values is shown in Supplementary Table 6. It is important to note that these values represent the features most predictive of mental health in this model, but do not necessarily indicate whether they are risk factors or protective factors. To further elucidate the relationship between risk factors and mental health outcomes, a more information-dense summary relating the data-set features, SHAP values and model output is shown below in the beeswarm plot in Fig. 2. Beeswarm plots are useful as they display both the relative importance of values and their relationship to the predicted outcome. For categorical variables, higher feature values correspond to the category assumed to have the highest association with diagnosis of a mental health problem (e.g. higher feature value for parents’ smoking status would be ‘smoker’). Higher feature values are purple in colour and lower feature values are orange in colour. Each individual data point represents the SHAP value for a specific variable for one child in the data-set. Positive data points further to the right on the *x*-axis represent SHAP values more strongly associated with a mental health problem.

Figure 2 suggests that our previous intuition regarding risk factors was accurate for most risk factors, including looked after child status: looked after, substance misuse: misusing substances and school exclusion category: fixed term exclusion, which validates these as important mental health risk factors. Being a boy and intermediate age (e.g. 13–15 years old) were also risk factors. Other factors associated with having a mental health problem that are consistent with previous studies were the presence of mental health problems in parents, a diagnosis of autism and living in a region with a higher index of multiple deprivation. Some presumed risk factors, such as the absence of care for dental checks and immunisations, were associated with a lower risk of mental health problems. Two ICD-10 codes: Z9 (‘Persons with potential health hazards related to family and personal history and certain conditions influencing health status’) and T3 (‘Burns and corrosions of multiple and unspecified body; Frostbite’) were associated with mental health problems.

### Algorithmic fairness

**Fig. 3:**
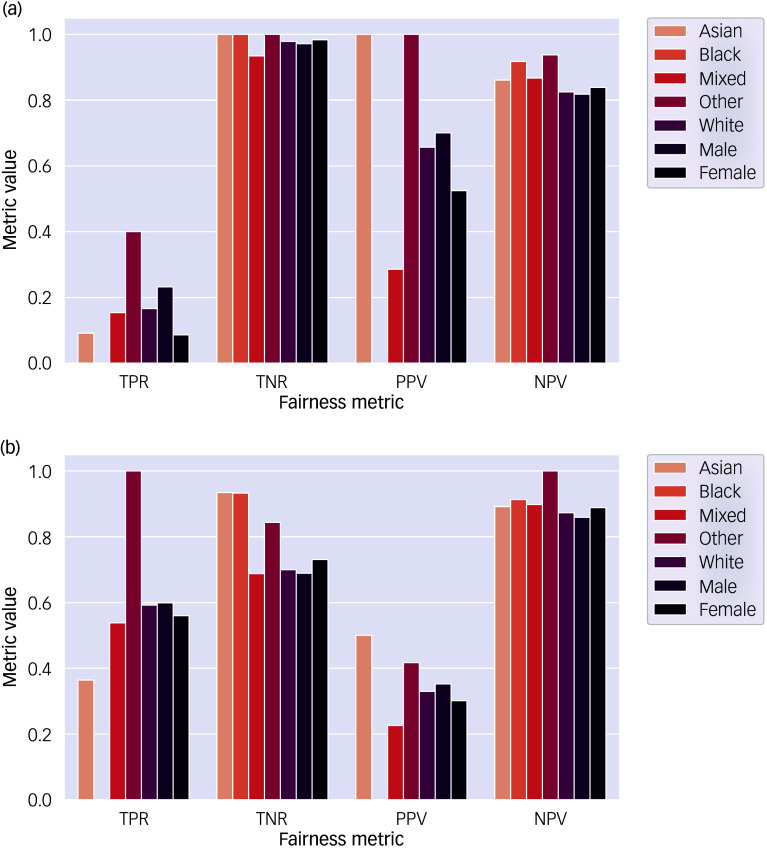
Assessment of algorithmic fairness. (a) Gradient boosting classifier model. (b) Logistic regression model. NPV, negative predictive value; PPV, positive predictive value; TNR, true negative rate (specificity); TPR, true positive rate (sensitivity).

For the best-performing model (gradient boosting classifier) and logistic regression baseline model, assessments of algorithmic fairness are displayed in Fig. 3, using an 80–20 train-test split. These results in tabular form can be seen in Supplementary Table 7. For all of these fairness metrics, values closer to 1 signify better performance, whereas values closer to 0 signify worse performance.

The gradient boosting classifier had a high specificity (TNR) and low sensitivity (TPR) for all ethnic and gender groups, indicating that it was better at correctly predicting individuals who did not have a mental health problem and had more difficulty correctly predicting individuals with a mental health problem. In contrast, the logistic regression model more effectively balanced the two classes, with similar results for sensitivity (TPR) and specificity (TNR). The gradient boosting classifier exhibited similar specificity (TNR) and NPV performance for all ethnicities and biological gender, but showed more variance in sensitivity (TPR) and PPV with a sensitivity of 0 for Black children. In both models, there was possible gender bias, with a higher sensitivity (TPR) and PPV for boys and a higher specificity (TNR) and NPV for girls.

## Discussion

### Main findings

Within the cohort, there was a higher prevalence of mental health problems compared with the general population of children in the UK. This could reflect that children in social care experience more ACEs, known to be associated with a range of mental health issues.^33^ The models predicting mental health problems achieved good performance on cross-validation analysis. However, the models showed significant bias in their ability to predict accurately in subgroups. Both the logistic regression and gradient boosting models were less able to predict mental health problems in girls and Black, Asian and mixed ethnicity children. Given the known disadvantage these groups already experience in accessing mental health services, it would be critical that a prediction tool not exacerbate these difficulties. Additionally, the models were less able to identify mental health problems than predict those not experiencing mental health problems. This may reflect sample size and data-set imbalance. However, given the difficulty and inconsistency in identification of cases in this cohort already, it is important that any models taken forward should further refine classification thresholds to increase identification of mental health problems. The models developed require refinement, including exploring relative performance in important subgroups such as specific mental health conditions.

The two main analysis metrics (AUROC and AUPRC) both pointed toward the superior performance of some machine learning models, with high concordance between the two metrics. As expected, the AUPRC was lower than the AUROC for all models assessed. Prior research has demonstrated that machine learning models perform no better than logistic regression classifiers.^34^ In contrast, our study found that three machine learning models (random forest, SVM and gradient boosting classifier) slightly outperformed the traditional logistic regression model by some measures such as AUROC and AUPRC. This improvement in overall performance could reflect non-linear relationships within the data that are best modelled with non-linear methods. However, the differences in model performance were relatively small and fall within overlapping confidence intervals, and by some algorithmic fairness metrics, the regression models were superior. Additionally, the logistic regression model performed marginally better than the MLP model, which was the most complex model assessed.

By linkage of a range of data-sets that included health, social care and education information, we created a data-set containing a wide range of biopsychosocial risk factors that we hypothesised would be important to prediction. The risk factors contributing most to the models’ predictive performance were indeed heterogeneous, and included individual characteristics such as age, physical health (e.g. having a physical disability) and risk factors related to family (e.g. mental health problems of parents). Linkage of routinely collected data spanning childhood from health, education and social care sources is one important mechanism of accessing the data needed for mental health prediction. This process also aligned with several recommendations for minimising model bias, such as identifying candidate predictors *a priori* by using clinical experience, existing research evidence or previous models.^35^

Many of the risk factors most predictive of mental health problems were already well established, including substance misuse, having a physical disability and the presence of neurodiversity. Other mental health risk factors identified were less established and contribute to growing knowledge about mental health risk, such as school exclusions.^36^ Further, the association between some ICD-10 codes and mental health outcomes demonstrates the inextricable link between physical and mental health. Additionally, the presence of missing data for some features (e.g. health surveillance checks) was associated with the presence of a mental health problem. Missing data is difficult to interpret because observed changes could be attributable to individual-level factors or factors relating to data collection. It is possible that missing data for certain risk factors, such as child health surveillance checks, could be tied to patterns of service use. Further, many of the missing data features had Shapley values with wide confidence intervals, likely reflecting the small sample sizes of missing data classes and the heterogeneity of individuals with missing data. Additionally, contrary to the general literature, some presumed risk factors such as the absence of care for dental checks and immunisations were surprisingly associated with a lower risk of mental health problems. This is a specific cohort with social care contact, and these findings could be attributable to data-set idiosyncrasies, association with other protective factors or statistical noise, and merit further evaluation before implementation. Taken together, this evaluation of risk factor importance provides information on mental health risk factors for children in social care that researchers and clinicians should consider when prioritising data inclusion for mental health prediction models in the future.

Finally, it is imperative that models be rigorously assessed for algorithmic bias to ensure that they do not exacerbate existing health disparities,^37^ especially if models are to help decide resource allocation. We found model performance trends between ethnicities difficult to disentangle because of the small sample sizes for many ethnicities within the test set, despite using data from the entirety of Welsh children with social care contact. Nonetheless, it is notable that no Black children with a mental health diagnosis were correctly identified by the model. It will be important, moving forward, to continue to use responsible machine learning frameworks that integrate data from larger, more diverse data-sets with representation of underserved communities. For biological gender, both models were slightly more likely to identify mental health problems (i.e. true positives) in boys than girls, while more often identifying lack of mental health problems (i.e. true negatives) in girls than boys. This trend can be partially explained by the higher prevalence of mental health problems in boys (21.30%) than girls (18.43%) in the data-set. However, additional factors such as model bias are likely involved. Further evaluation of model fairness is necessary to ensure these models do not exacerbate healthcare disparities.

### Limitations

There are limitations relevant to both cohort creation and model development. The lack of timestamp granularity for social care data-sets in the SAIL Databank prohibited us from modelling the data by using time-series approaches, which could have improved performance. More granular information about the point at which exposure takes place may be one way to improve the predictive performance of models. Further, the SAIL Databank metadata was sometimes ambiguous, forcing us to omit otherwise useful indicators such as urbanicity. Moreover, by focusing solely on children with social care encounters in Wales, generalisability to other populations is diminished. However, this work may be useful for the Welsh population, and can still serve as an effective guide when developing more generalisable models.

Importantly, some children with mental health problems either do not seek support services or are unable to access them,^38^ and consequently cannot be identified with this paper’s methods. Thus, through this analysis, we are not able to necessarily identify all children with mental health problems, only children with mental health problems that are identified by services. This is partially mitigated by these children being in social care and, thus, having increased monitoring and some access to mental health services. Further exacerbating biases, outcome labels are likely skewed toward mental health problems for individuals with more severe mental health problems. Our cohort also lacked detailed parental information, which may limit access to important risk factors. For example, in a similar study, 72.3% of ACEs were found in maternal records.^39^ A final modelling limitation was our inability to perform an external validation of model performance. Although we applied proper internal validation safeguards on a large data-set, external validations remain the gold standard in model development.

### Implications and future work

This work comprises one portion of the overarching Timely project, which aims to create early identification tools for childhood mental health problems. The team is creating CADRE (Child and Adolescent Data Resource; www.cadre.org.uk), a database containing longitudinal administrative data relating to health, social care and education for young people. The aim is for CADRE to support real-time clinical decision-making, with a de-identified version available to approved researchers. CADRE will form part of a network of Trusted Research Environments that can utilise genetic data and will include unstructured data such as anonymised clinical notes in addition to routinely-collected data on health, education and social care. The models prototyped within the present work described here will be refined and externally validated in the CADRE database.

There is scant prior work using predictive modelling to identify general mental health problems in children, with a recent systematic review^40^ finding only two articles meeting these criteria.^9,41^ Although difficult to directly compare results, especially since our cohort of children all had social care contact, our model performance here is on par with previous studies with data-sets curated specifically for mental health prediction. This work also builds upon these previous analyses by assessing a substantially larger cohort of 26 820 children (whereas the prior two studies looked at 7638 and 60 children). In this work, we additionally identified mental health risk factors that healthcare professionals should consider when caring for children, especially those with social care contact. Finally, this analysis also details machine learning techniques including assessments of algorithmic fairness useful for future related work. Collectively, this work marks a step toward equitable and effective machine learning prediction of childhood mental health problems.

## Supporting information

Crowley et al. supplementary material Crowley et al. supplementary material

## Data Availability

The trained logistic regression model can be found at https://github.com/ryan3741/SAIL-ML-Model. The other models are not available currently due to the possibility of machine learning model parameters of more complex models inadvertently containing sensitive data. The raw data and analytical code used for this study are housed by the SAIL Databank. The SAIL Databank is not available publicly, but researchers can access the data following approval by the SAIL Information Governance Review Panel. Information regarding this application process can be found at https://saildatabank.com/data/apply-to-work-with-the-data/. The initial study protocol can be found at https://whatworks-csc.org.uk/wp-content/uploads/Final_Protocol_Cambridge_Spark.pdf. There was no patient or public involvement in the design of this study.

## Supplementary material

The supplementary material for this article can be found at https://doi.org/10.1192/bjo.2025.32
