# Supplementary material for: Machine learning for prediction of childhood mental health problems in social care
Source: BJPsych Open. 2025 Apr 11;11(3):e86. doi: 10.1192/bjo.2025.32 (PMC12052593; doi:10.1192/bjo.2025.32)
Supplement: Crowley et al. supplementary material [file S2056472425000328sup001.docx]

**Appendix**

**Table 1: TRIPOD+AI Checklist**

TRIPOD+AI Checklist

| **Section/Topic Item Dev/Eval Checklist item** | | | | | **Page of original manuscript** |
| --- | --- | --- | --- | --- | --- |
| **TITLE** | | | | |  |
| *Title* | 1 | | D;E | Identify the study as developing or evaluating the performance of a multivariable prediction model, the target population, and the outcome to be predicted | Title page |
| **ABSTRACT** | | | | | |
| *Abstract* | 2 | | D;E | See TRIPOD+AI for Abstracts checklist | NA |
| **INTRODUCTION** | | | | | |
| *Background* | 3a | | D;E | Explain the healthcare context (including whether diagnostic or prognostic) and rationale for developing or evaluating the prediction model, including references to existing models | 2-3 |
|  | 3b | | D;E | Describe the target population and the intended purpose of the prediction model in the context of the care pathway, including its intended users (e.g., healthcare professionals, patients, public) | 2-3 |
|  | 3c | | D;E | Describe any known health inequalities between sociodemographic groups | NA |
| *Objectives* | 4 | | D;E | Specify the study objectives, including whether the study describes the development or validation of a prediction model (or both) | 3 |
| **METHODS** | | | | | |
| *Data* | 5a | | D;E | Describe the sources of data separately for the development and evaluation datasets (e.g., randomized trial, cohort, routine care or registry data), the rationale for using these data, and representativeness of the data | 4-7 |
|  | 5b | | D;E | Specify the dates of the collected participant data, including start and end of participant accrual; and, if applicable, end of follow-up | 4 |
| *Participants* | 6a | | D;E | Specify key elements of the study setting (e.g., primary care, secondary care, general population) including the number and location of centres | 4 |
|  | 6b | | D;E | Describe the eligibility criteria for study participants | 4 |
|  | 6c | | D;E | Give details of any treatments received, and how they were handled during model development or evaluation, if relevant | NA |
| *Data preparation* | 7 | | D;E | Describe any data pre-processing and quality checking, including whether this was similar across relevant sociodemographic groups | 4-7 |
| *Outcome* | 8a | | D;E | Clearly define the outcome that is being predicted and the time horizon, including how and when assessed, the rationale for choosing this outcome, and whether the method of outcome assessment is consistent across sociodemographic groups | 4 |
|  | 8b | | D;E | If outcome assessment requires subjective interpretation, describe the qualifications and demographic characteristics of the outcome assessors | NA |
|  | 8c | | D;E | Report any actions to blind assessment of the outcome to be predicted | NA |
| *Predictors* | 9a | | D | Describe the choice of initial predictors (e.g., literature, previous models, all available predictors) and any pre-selection of predictors before model building | 4-6 |
|  | 9b | | D;E | Clearly define all predictors, including how and when they were measured (and any actions to blind assessment of predictors for the outcome and other predictors) | 4-6, Table S4 |
|  | 9c | | D;E | If predictor measurement requires subjective interpretation, describe the qualifications and demographic characteristics of the predictor assessors | NA |
| *Sample size* | 10 | | D;E | Explain how the study size was arrived at (separately for development and evaluation), and justify that the study size was sufficient to answer the research question. Include details of any sample size calculation | NA, all children meeting inclusion criteria studied |
| *Missing data* | 11 | | D;E | Describe how missing data were handled. Provide reasons for omitting any data | 5-6 |
| *Analytical methods* | 12a | | D | Describe how the data were used (e.g., for development and evaluation of model performance) in the analysis, including whether the data were partitioned, considering any sample size requirements | 4 |
|  | 12b | | D | Depending on the type of model, describe how predictors were handled in the analyses (functional form, rescaling, transformation, or any standardisation). | 5-6 |
|  | 12c | | D | Specify the type of model, rationale^2^, all model-building steps, including any hyperparameter tuning, and method for internal validation | 6-7 |
|  | 12d | | D;E | Describe if and how any heterogeneity in estimates of model parameter values and model performance was handled and quantified across clusters (e.g., hospitals, countries). See TRIPOD-Cluster for additional considerations^3^ | NA |
|  | 12e | | D;E | Specify all measures and plots used (and their rationale) to evaluate model performance (e.g., discrimination, calibration, clinical utility) and, if relevant, to compare multiple models | 7 |
|  | 12f | | E | Describe any model updating (e.g., recalibration) arising from the model evaluation, either overall or for particular sociodemographic groups or settings | NA |
|  | 12g | | E | For model evaluation, describe how the model predictions were calculated (e.g., formula, code, object, application programming interface) | NA |
| *Class imbalance* | 13 | | D;E | If class imbalance methods were used, state why and how this was done, and any subsequent methods to recalibrate the model or the model predictions | 6-7 |
| *Fairness* | 14 | | D;E | Describe any approaches that were used to address model fairness and their rationale | 7 |
| *Model output* | 15 | | D | Specify the output of the prediction model (e.g., probabilities, classification). Provide details and rationale for any classification and how the thresholds were identified | 6 |
| *Training versus*  *evaluation* | | 16 | D;E | Identify any differences between the development and evaluation data in healthcare setting, eligibility  criteria, outcome, and predictors | NA |
| *Ethical approval* | | 17 | D;E | Name the institutional research board or ethics committee that approved the study and describe the participant-informed consent or the ethics committee waiver of informed consent | 7 |
| **OPEN SCIENCE** | | | | | |
| *Funding* | | 18a | D;E | Give the source of funding and the role of the funders for the present study | 20 |
| *Conflicts of interest* | | 18b | D;E | Declare any conflicts of interest and financial disclosures for all authors | 20 |
| *Protocol* | | 18c | D;E | Indicate where the study protocol can be accessed or state that a protocol was not prepared | 19 |
| *Registration* | | 18d | D;E | Provide registration information for the study, including register name and registration number, or state  that the study was not registered | NA |
| *Data sharing* | | 18e | D;E | Provide details of the availability of the study data | 19 |
| *Code sharing* | | 18f | D;E | Provide details of the availability of the analytical code^4^ | 19 |
| **PATIENT & PUBLIC INVOLVEMENT** | | | | | |
| *Patient & Public Involvement* | | 19 | D;E | Provide details of any patient and public involvement during the design, conduct, reporting, interpretation, or dissemination of the study or state no involvement. | 19 |
| **RESULTS** | | | | | |
| *Participants* | | 20a | D;E | Describe the flow of participants through the study, including the number of participants with and without the outcome and, if applicable, a summary of the follow-up time. A diagram may be helpful. | 8-10 |
|  |  | 20b | D;E | Report the characteristics overall and, where applicable, for each data source or setting, including the key dates, key predictors (including demographics), treatments received, sample size, number of outcome events, follow-up time, and amount of missing data. A table may be helpful. Report any differences across key demographic groups. | 8-10 |
|  |  | 20c | E | For model evaluation, show a comparison with the development data of the distribution of important predictors (demographics, predictors, and outcome). | NA |
| *Model development* | | 21 | D;E | Specify the number of participants and outcome events in each analysis (e.g., for model development, hyperparameter tuning, model evaluation) | 8-10 |
| *Model specification* | | 22 | D | Provide details of the full prediction model (e.g., formula, code, object, application programming interface) to allow predictions in new individuals and to enable third-party evaluation and implementation, including any restrictions to access or re-use (e.g., freely available, proprietary)^5^ | 19 |
| *Model performance* | | 23a | D;E | Report model performance estimates with confidence intervals, including for any key subgroups (e.g., sociodemographic). Consider plots to aid presentation. | 10-11 |
|  |  | 23b | D;E | If examined, report results of any heterogeneity in model performance across clusters. See TRIPOD Cluster for additional details^3^. | NA |
| *Model updating* | | 24 | E | Report the results from any model updating, including the updated model and subsequent performance | NA |
| **DISCUSSION** | | | | | |
| *Interpretation* | | 25 | D;E | Give an overall interpretation of the main results, including issues of fairness in the context of the objectives and previous studies | 15-19 |
| *Limitations* | | 26 | D;E | Discuss any limitations of the study (such as a non-representative sample, sample size, overfitting, missing data) and their effects on any biases, statistical uncertainty, and generalizability | 18-19 |
| *Usability of the model in the context of current care* | | 27a | D | Describe how poor quality or unavailable input data (e.g., predictor values) should be assessed and handled when implementing the prediction model | NA- models were not developed for deployment |
|  |  | 27b | D | Specify whether users will be required to interact in the handling of the input data or use of the model, and what level of expertise is required of users | NA |
|  |  | 27c | D;E | Discuss any next steps for future research, with a specific view to applicability and generalizability of the model | 19 |

From: Collins GS, Moons KGM, Dhiman P, et al. *BMJ* 2024;385:e078378. doi:10.1136/bmj-2023-078378 models were not develop

^1^ D=items relevant only to the development of a prediction model; E=items relating solely to the evaluation of a prediction model; D;E=items applicable to both the development and evaluation of a prediction model

^2^ Separately for all model building approaches.

^3^ TRIPOD-Cluster is a checklist of reporting recommendations for studies developing or validating models that explicitly account for clustering or explore heterogeneity in model performance (eg, at different hospitals or centres). Debray et al, BMJ 2023; 380: e071018 [DOI: 10.1136/bmj-2022-071018]

^4^ This relates to the analysis code, for example, any data cleaning, feature engineering, model building, evaluation.

^5^ This relates to the code to implement the model to get estimates of risk for a new individual.

**Table 2: SAIL Datasets**

| **Group Name** | **Individual Data Sources** | **Included in Final Analysis** |
| --- | --- | --- |
| Demographics | Annual District Birth Extract (ADBE)    Annual District Death Extract (ADDE)  Welsh Demographic Service Dataset (WDSD) | No  No  Yes |
| Education | Pre16 Education Attainment (EDUW) | Yes |
| General Practice | GP Primary Care – Audit (WLGP) | No |
| Healthcare | Critical Care Dataset (CCDS)  Emergency Department Dataset (EDDS)    Maternity Indicators Dataset (MIDS)  National Community Child Health (NCCH)    NHS Hospital Outpatients (OPDW)  NHS 111 Call data (NHSO)    Outpatient Referrals from Primary Care (OPRD)  Patient Episode Database for Wales (PEDW)  Substance Misuse Dataset (SMDS)    Wales Results Reporting Service (WRRS) | No  No  Yes  Yes  No  No  No  Yes  No  No |
| Social Care | Child In Need Wales (CINW)    Children Receiving Care and Support (CRCS)    Looked After Children Wales (LACW) | Yes  Yes  No |

**Table 3a: Risk Factors from Delphi Framework (Social and Environmental Domain)**

| **Risk Factor** | **Level** |
| --- | --- |
| Air pollution | Society |
| Area deprivation (area code) | Society |
| Ethnic minority in low ethnic density area | Society |
| English as an additional language | Society |
| Gangland crime | Society |
| Remoteness of living | Society |
| Urbanicity | Society |
| War/conflict | Society |
| Broken or complex family structure | Family/caregiver |
| Death of close relative (excluding primary caregiver(s)) | Family/caregiver |
| Death of primary caregiver(s) | Family/caregiver |
| Domestic violence | Family/caregiver |
| Family conflict or discord | Family/caregiver |
| Family financial problems | Family/caregiver |
| Famine/food poverty | Family/caregiver |
| High socioeconomic status family | Family/caregiver |
| Household alcohol abuse | Family/caregiver |
| Household criminality | Family/caregiver |
| Household drug abuse | Family/caregiver |
| Household mental illness | Family/caregiver |
| Household overcrowding | Family/caregiver |
| Low social support for family | Family/caregiver |
| Low socioeconomic status family | Family/caregiver |
| Multi-generational families within the same home | Family/caregiver |
| Number of living children in the family | Family/caregiver |
| Poor primary caregiver-caregiver relationship | Family/caregiver |
| Poor relationship between primary caregivers | Family/caregiver |
| Primary caregiver(s) marital status | Family/caregiver |
| Primary caregiver(s) parenting styles (strict/rigid/conventional) | Family/caregiver |
| Primary caregiver(s) unemployment | Family/caregiver |
| Single parent/caregiver family | Family/caregiver |
| Smoking status of primary caregiver(s) | Family/caregiver |
| Smoking status of primary caregiver(s) | Family/caregiver |
| Being a second generation immigrant | Individual |
| Being a young carer | Individual |
| Child younger than classmates | Individual |
| Chronic psychological stress | Individual |
| Cumulative traumatic events | Individual |
| Death of a close friend | Individual |
| Emotional neglect | Individual |
| Emotional, psychological or verbal abuse | Individual |
| Ethnicity | Individual |
| Experience of past traumatic event | Individual |
| Frequent fear of family member | Individual |
| Experience of racism and discrimination | Individual |
| Injury during a traumatic event | Individual |
| Maternal alcohol use during pregnancy | Individual |
| Maternal smoking during pregnancy | Individual |
| Maternal substance abuse during pregnancy | Individual |
| Maternal use of psychotropics during pregnancy | Individual |
| Month of birth | Individual |
| Not breast fed | Individual |
| Perceived pressure to be thin | Individual |
| Physical abuse | Individual |
| Physical neglect | Individual |
| Poor online peer relationships | Individual |
| Poor peer relationships | Individual |
| Poor primary caregiver-child relationship | Individual |
| Separation from family (e.g. out-of-home care) | Individual |
| Sexual abuse | Individual |
| Torture | Individual |
| Trapped during earthquake | Individual |
| Trauma severity | Individual |
| Victim of bullying | Individual |
| Victim or witness of violent crime | Individual |
| Witness of community violence | Individual |
| Witnessing injury/death during a traumatic event | Individual |

**Table 3b: Risk Factors from Delphi Framework (Behavioural Domain)**

| **Risk Factor** | **Level** |
| --- | --- |
| Physical inactivity in family/primary care-givers | Family/caregiver |
| Unhealthy diet in family/primary care-givers | Family/caregiver |
| Diet low in plant-matter | Individual |
| Diet low in plant-matter diversity | Individual |
| Excessive physical activity | Individual |
| Excessive sports participation | Individual |
| Foods high in trans fats in diet | Individual |
| Frequent social media use | Individual |
| Heavy alcohol use | Individual |
| High ratio of omega-6 to omega-3 fatty acids in diet | Individual |
| Higher behavioural inhibition | Individual |
| Lower behavioural inhibition | Individual |
| Low levels of omega-3 polyunsaturated fatty acids in diet | Individual |
| Low sports participation | Individual |
| Nitrous oxide use | Individual |
| Non-prescription drug use | Individual |
| Physical inactivity | Individual |
| Poor sleep patterns | Individual |
| Prescription drug abuse | Individual |
| Sexual risk-taking | Individual |
| Smoking | Individual |
| High levels of ultra-processed foods in diet | Individual |
| Unhealthy diet | Individual |

**Table 3c: Risk Factors from Delphi Framework (Education/Employment Domain)**

| **Risk Factor** | **Level** |
| --- | --- |
| Increased pressure/stress for teachers (e.g. insufficient pay/resources and poor leadership) | Society |
| Poor school climate (e.g. Ofsted weightings) | Society |
| Poor quality of a lack of social-emotional learning programmes in pre-school | Society |
| Quality of school climate (e.g. relating to school connectedness, feelings of safety in school, perception of school, adult-student relationships, morale) | Society |
| School composition (e.g. size/headcount, gender proportions, ethnicity proportions) | Society |
| School-level deprivation (e.g. proportion eligible for free school meals) | Society |
| Language impairment in primary caregivers | Family/caregiver |
| Low education level of caregiver(s) (other than primary caregiver(s)) | Family/caregiver |
| Low education level of primary caregiver(s) | Family/caregiver |
| Child not read to daily by age 1 | Individual |
| Disruptive behaviours in school (e.g. defiance and non-compliance) | Individual |
| Having an Education and Health Care Plan (EHCP) | Individual |
| Lack of imitation games with caregivers by age 1 | Individual |
| Out-of-school discipline (e.g. suspension and expulsion) | Individual |
| Participation in the Free-Lunch Program | Individual |
| Poor educational attainment | Individual |
| Poor peer relationships | Individual |
| Poor school attendance | Individual |
| Poor teacher-pupil relationship | Individual |
| School exclusions | Individual |
| Special Educational Needs (SEN) | Individual |
| Unemployment of the individual | Individual |

**Table 3d: Risk Factors from Delphi Framework (Biomarkers Domain)**

| **Risk Factor** | **Level** |
| --- | --- |
| Age of parents at time of giving birth | Individual |
| Birth length | Individual |
| Birth weight | Individual |
| CD14 levels | Individual |
| Changes in amygdala (e.g. reduced volume) | Individual |
| Changes in prefrontal cortex (e.g. reduced total volume; low myelination; low white matter; increased middle inferior and ventral regions and superior/dorsal regions) | Individual |
| Chronic inflammation | Individual |
| Decreased neuronal branching | Individual |
| Epigenetic marker - chromatin regulation | Individual |
| Epigenetic marker - DNA methylation | Individual |
| Epigenetic marker - non-coding ribonucleic acids | Individual |
| Genetic markers | Individual |
| Gut microbiome (reduced diversity and reduced populations of certain bacteria) | Individual |
| High intelligence quotient (IQ) | Individual |
| High levels of cytokine proteins (e.g. interleukin-1 [IL-1]; interleukin-6 [IL-6]) | Individual |
| High levels of indoleamine-2,3-dioxygenase (IDO) | Individual |
| High levels of tryptophan-2,3-dioxygenase (TDO) | Individual |
| High peripheral manganese | Individual |
| High permeability of blood-brain barrier | Individual |
| HPA axis and glucocorticoid receptor resistance | Individual |
| Increased levels of chemokines | Individual |
| Increased levels of eicosanoids | Individual |
| Increased neurodegeneration | Individual |
| Increased quinolinic acid (QUIN) | Individual |
| Kynurenine/tryptophan ratio | Individual |
| Low levels of dopamine | Individual |
| Low or high levels of GDNF (i.e. neurotrophic factor) | Individual |
| Low or high levels of Tumour Necrosis Factor alpha (TNF-α) | Individual |
| Low intelligence quotient (IQ) | Individual |
| Low levels of B vitamins | Individual |
| Low levels of folate | Individual |
| Low levels of melatonin | Individual |
| Low levels of nutrients | Individual |
| Low levels of serotonin | Individual |
| Low levels of vitamin D | Individual |
| Low serum ferritin | Individual |
| Neuronal atrophy | Individual |
| Neurotoxicity | Individual |
| Neutrophil–lymphocyte ratio (NLR) | Individual |
| Nutritional deficits | Individual |
| Raised C-reactive protein (CRP) | Individual |
| Raised levels of glucocorticoids/cortisol | Individual |
| Reduced grey matter in hippocampus | Individual |
| Reduced kynurenic acid (KYNA) | Individual |
| Reduced levels of BDNF (i.e. neurotrophic factor) | Individual |
| Reduced neurogenesis | Individual |
| Reduced neuroplasticity | Individual |
| Sex (biological) | Individual |

**Table 3e: Risk Factors from Delphi Framework (Physical Health Domain)**

| **Risk Factor** | **Level** |
| --- | --- |
| Family history of autoimmune disease | Family/caregiver |
| Family history of psoriasis | Family/caregiver |
| Family history of rheumatoid arthritis | Family/caregiver |
| Family history of Type 1 diabetes | Family/caregiver |
| Maternal self-rated health (prior to pregnancy) | Family/caregiver |
| Primary caregiver(s) chronic (long lasting) illness | Family/caregiver |
| Severe illness in family | Family/caregiver |
| 5-min Apgar score <7 | Individual |
| Allergies (e.g. non-lgE-mediated food allergies, pollen allergies) | Individual |
| Anaemia | Individual |
| Autoimmune disorders (e.g. rheumatoid arthritis) | Individual |
| Asthma | Individual |
| Body mass Index (BMI) | Individual |
| Eczema | Individual |
| Chronic (long lasting) gastric ill-health (e.g. inflammatory bowel disease) | Individual |
| Chronic (long lasting) infection (e.g. Lyme disease, periodontal disease) | Individual |
| Chronic (long lasting) reflux or indigestion | Individual |
| Congenital malformations | Individual |
| Dental caries (tooth decay) | Individual |
| Dental erosion | Individual |
| Diabetes | Individual |
| Disease history | Individual |
| Global developmental delay | Individual |
| Hypoxia (at birth) | Individual |
| Inflammatory diseases (e.g. Lyme disease) | Individual |
| Irritable Bowel Syndrome (IBS) | Individual |
| Lack of response to treatment for a physical health condition | Individual |
| Learning disability | Individual |
| Long term antibiotic use | Individual |
| Low diversity & composition of gut microbiota | Individual |
| Low serum vitamin D | Individual |
| Maternal hypertension during pregnancy | Individual |
| Maternal infection requiring hospitalisation during pregnancy | Individual |
| Maternal obesity during pregnancy | Individual |
| Maternal acetaminophen (paracetamol) use during pregnancy | Individual |
| Maternal auto-immune disease during pregnancy | Individual |
| Maternal diabetes during pregnancy | Individual |
| Maternal obesity/overweight during pregnancy | Individual |
| Obesity/overweight | Individual |
| Obstetric complications (other) | Individual |
| Perinatal infections (e.g. cytomegalovirus) | Individual |
| Polyhydramnios | Individual |
| Pre-eclampsia | Individual |
| Premature birth | Individual |
| Prolonged duration of a physical health condition | Individual |
| Repeated infections | Individual |
| Ruptured membranes (at birth) | Individual |
| Severe health condition | Individual |
| Sleep disorder | Individual |
| Thyroid disease | Individual |
| Traumatic brain injury | Individual |

**Table 3f: Risk Factors from Delphi framework (Psychological and Mental Health Domain)**

| **Risk Factor** | **Level** |
| --- | --- |
| Negative attitudes to mental health problems within society an individual lives in | Society |
| Family history of psychiatric disorders | Family/caregiver |
| Family history of severe mental illness (e.g. psychosis) | Family/caregiver |
| Primary caregiver(s) mental health problems | Family/caregiver |
| Acute stress disorder (as a predictor of further mental health problems) | Individual |
| Acute stress symptoms (anxiety, avoidance or depression -as a predictor of further mental health problems) | Individual |
| Anxiety (as a predictor of further mental health problems) | Individual |
| Attention (as a predictor of further mental health problems) | Individual |
| Dissociation during traumatic experience | Individual |
| Emotional reactivity | Individual |
| Excessive rumination | Individual |
| High levels of perceived stress | Individual |
| Increased anxiety arousal | Individual |
| Increased panic attacks | Individual |
| Ineffective coping strategies | Individual |
| Lack of psychological resilience | Individual |
| Maternal depression during pregnancy | Individual |
| Maternal psychosis (perinatal or pre-natal) | Individual |
| Poor concentration | Individual |
| Poor problem-solving abilities | Individual |
| Poor visuospatial functioning | Individual |
| Problems with memory | Individual |
| Psychiatric history | Individual |

**Table 3g: Risk Factors from Delphi framework (Patterns of Service Use)**

| Lack of attendance to dental visits | Patterns of Service Use | Individual |
| --- | --- | --- |
| Missed ante-natal visits | Patterns of Service Use | Individual |
| Missed doctor appointments | Patterns of Service Use | Individual |
| Missed postnatal visits | Patterns of Service Use | Individual |
| Missed routine check-up appointments | Patterns of Service Use | Individual |
| Missed vaccination appointments | Patterns of Service Use | Individual |
| Presentation to multiple services (e.g. different hospitals, GPs, social care etc) | Patterns of Service Use | Individual |
| Repeat hospitalisation | Patterns of Service Use | Individual |

**Table 3h: Risk Factors from Delphi Framework (Factors Relevant to Under-Served Populations Domain)**

| **Risk Factor** | **Level** |
| --- | --- |
| Area deprivation (area code) | Society |
| English as an additional language | Society |
| Ethnic minority in low ethnic density area | Society |
| Gangland crime | Society |
| Increased pressure/stress for teachers (e.g. insufficient pay/resources and poor leadership) | Society |
| Poor school climate (e.g. Ofsted weightings) | Society |
| Quality of school climate (e.g. relating to school connectedness, feelings of safety in school, perception of school, adult-student relationships, morale) | Society |
| School composition (e.g. size/headcount, gender proportions, ethnicity proportions) | Society |
| School-level deprivation (e.g. proportion eligible for free school meals) | Society |
| War/conflict | Society |
| Family financial problems | Family/caregiver |
| Family experience of social exclusion, discrimination and harassment associated with ethnicity | Family/caregiver |
| Famine/food poverty | Family/caregiver |
| Household overcrowding | Family/caregiver |
| Low socioeconomic status | Family/caregiver |
| Multi-generational families within the same home | Family/caregiver |
| Primary caregiver(s) parenting styles (strict/rigid/conventional) | Family/caregiver |
| Primary caregiver(s) unemployment | Family/caregiver |
| Being a second generation immigrant | Individual |
| Being a young carer | Individual |
| English as an additional language | Individual |
| Ethnicity | Individual |
| Experience of bereavement during a traumatic event | Individual |
| Experience of racism and discrimination | Individual |
| Participation in the Free-Lunch Program | Individual |
| Trapped during earthquake | Individual |
| Unemployment | Individual |

**Table 4: Delphi Risk Factors and Inclusion in Final Model**

| **Risk Factor** | **Included in Final Model*** |
| --- | --- |
| 5-min Apgar score <7 | Yes |
| Acute stress disorder (as a predictor of further mental health problems) | No |
| Acute stress symptoms (anxiety, avoidance or depression - as a predictor of further mental health problems) | No |
| Age of parents at time of giving birth | Yes |
| Allergies (e.g. non-lgE-mediated food allergies, pollen allergies) | No |
| Anaemia | No |
| Anxiety (as a predictor of further mental health problems) | No |
| Area deprivation (area code) | Yes |
| Asthma | No |
| Autoimmune disorders (e.g. rheumatoid arthritis) | No |
| Being a looked after child (LAC) | Yes |
| Being a young parent | No |
| Being an unaccompanied asylum seeker | Yes |
| Birth length | No |
| Birth weight | Yes |
| Body mass Index (BMI) | No |
| Broken or complex family structure | Yes |
| Child in need (CIN) status | No |
| Child protection record | Yes |
| Child younger than classmates | Yes |
| Chronic (long lasting) gastric ill-health (e.g. inflammatory bowel disease) | No |
| Chronic (long lasting) infection (e.g. Lyme disease, periodontal disease) | No |
| Chronic (long lasting) reflux or indigestion | No |
| Chronic inflammation | No |
| Congenital malformations | No |
| Diabetes | No |
| Disease history | Yes |
| Domestic violence | Yes |
| Eczema | No |
| Emotional neglect | No |
| Emotional, psychological or verbal abuse | Yes |
| Ethnicity | Yes |
| Experiencing financial abuse | No |
| Failure to attend three or more planned health or social care appointments | No |
| Family conflict or discord | Yes |
| Family financial problem | Yes |
| Family history of psychiatric disorders | No |
| Family history of severe mental illness (e.g. psychosis) | No |
| Global developmental delay | No |
| Hearing impairment causing disability (e.g. deafness) | No |
| Heavy alcohol use | No |
| Homelessness | No |
| Homelessness (young person has left home) | No |
| Household alcohol abuse | No |
| Household drug abuse | No |
| Household mental illness | No |
| Hypoxia (at birth) | No |
| Increased panic attacks | No |
| Inflammatory diseases (e.g. Lyme disease) | No |
| Injury during a traumatic event | Yes |
| Intellectual disability | No |
| Investigations by multiple services suggestive of suffering from medically unexplained symptoms | No |
| Involvement in criminal justice system | No |
| Irritable Bowel Syndrome (IBS) | No |
| Lack of attendance to dental visits | Yes |
| Learning disability | Yes |
| Long term antibiotic use | No |
| Low education level of caregiver(s) (other than primary caregiver(s)) | Yes |
| Low levels of B vitamins | No |
| Low levels of folate | No |
| Low levels of vitamin D | No |
| Low serum ferritin | No |
| Low serum vitamin D | No |
| Low socioeconomic status | Yes |
| Lower behavioural inhibition | Yes |
| Maternal depression during pregnancy | No |
| Maternal obesity/overweight during pregnancy | No |
| Maternal smoking during pregnancy | No |
| Maternal substance abuse during pregnancy | Yes |
| Missed routine check-up appointments | Yes |
| Missed vaccination appointments | Yes |
| Month of birth | No |
| Neuro-developmental conditions (e.g. autism) | Yes |
| Nitrous oxide use | No |
| Non-prescription drug use | Yes |
| Not breast fed | Yes |
| Obesity/overweight | No |
| Out-of-school discipline (e.g. suspension and expulsion) | Yes |
| Participation in the Free-Lunch Program | Yes |
| Physical abuse | No |
| Physical disability | Yes |
| Physical neglect | Yes |
| Poor educational attainment | No |
| Poor school attendance | No |
| Premature birth | Yes |
| Prescription drug abuse | No |
| Primary caregiver(s) chronic (long lasting) illness | Yes |
| Primary caregiver(s) mental health problems | Yes |
| Primary caregiver(s) unemployment | No |
| Problems with memory | Yes |
| Prolonged duration of a physical health condition | Yes |
| Psychiatric history | No |
| Repeat hospitalisation | No |
| Repeated infections | No |
| School exclusions | Yes |
| School-level deprivation (e.g. proportion eligible for free school meals) | Yes |
| Serving in the military | No |
| Severe health condition | No |
| Severe illness in family | No |
| Sex (biological) | Yes |
| Sexual abuse | No |
| Sleep disorder | No |
| Smoking status of primary caregiver(s) | Yes |
| Special Educational Needs (SEN) | Yes |
| Three or more presentations to emergency services within a year | No |
| Thyroid disease | No |
| Traumatic brain injury | No |
| Unemployment of the individual | No |
| Urbanicity | No |
| Visual impairment causing disability (e.g. blindness/partial sightedness) | No |

********Risk factors with high degree of missingness (>80%) were not included in the final model*

**Table 5: Hyperparameter Tuning by Model**

| **Algorithm** | **Tuning Parameters*** |
| --- | --- |
| Logistic Regression | N/A |
| SVM (Radial Basis Function Kernel) | Cost (.1, .25, .5, **1**)  Gamma (2e-3, **2e-2** ,2e-1, 1) |
| Random Forest | Number of Estimators (50, 100, 150,**200**)  Max Tree Depth (1, 2, 3, **4**)  Max Features (4, 8, 12, **16**) |
| Gradient Boosting Classifier | Number Estimators (50, 100, **150**)  Max Depth (1, 2, 3, **4**)  Max Features (4, 8, 12, **16**) |
| MLP Classifier | Learning Rate (1e-4, 1e-3, 1e-2, **1e-1**)  Number of Hidden Layers (**2**, 4)  Number of Nodes Per Layer (**20**, 40, 80) |

********Bolded hyperparameter values reflect the optimal hyperparameters most commonly selected during hyperparameter tuning*

**Table 6: Comprehensive Mean Absolute Shapley Values for Gradient Boosting Classifier Model**

| **Risk Factor** | **Shapley Value (95% CI)** |
| --- | --- |
| Age: Continuous Value | .3201 (.2903 - .3509) |
| Apgar 1-Minute Score: Continuous Value | .0241 (.0094 - .0497) |
| Apgar 5-Minute Score: Continuous Value | .0375 (.0170 - .0668) |
| Asylum Seeker Status: Asylum Seeker | .011 (.0001 - .0038) |
| Asylum Seeker Status: Unknown | .0013 (.0003 - .0031) |
| Birth Weight: Continuous Value | .0277 (.0168 - .0440) |
| Breastfeed Status (8 weeks): Not Breastfed | .0122 (.0019 - .0340) |
| Breastfeed Status (8 weeks): Unknown | .0073 (.0010 - .022) |
| Breastfeed Status (Birth): Not Breastfed | .0120 (.0032 - .0297) |
| Breastfeed Status (Birth): Unknown | .0104 (.0034 - .0258) |
| Health Surveillance Checks Status: Not Receiving Checks | .0320 (.0068 - .0657) |
| Health Surveillance Checks Status: Unknown | .0916 (.0575 - .1311) |
| Child Protection Register Status: Registered | .0347 (.0095 - .0633) |
| Diagnosis Code: A0 (Intestinal Infectious Diseases) | .0057 (.0008 - .0172) |
| Diagnosis Code: B3 (Other Viral Diseases; Mycoses) | .0044 (.0011 - .0127) |
| Diagnosis Code: B9 (Sequelae of Infectious and Parasitic Diseases; Bacterial, Viral, and other Infectious Agents) | .0029 (.0004 - .0093) |
| Diagnosis Code: E8 (Metabolic Disorders) | .0037 (.0005 - .0113) |
| Diagnosis Code: G4 (Episodic and Paroxysmal Disorders) | .0037 (.0008 - .0089) |
| Diagnosis Code: G8 (Cerebral Palsy and other Paralytic Syndromes) | .0088(.0018 - .0205) |
| Diagnosis Code: H5 (Disorders of Ocular Muscles, Binocular Movement, Accommodation and Refraction; Visual Disturbances and Blindness; Other Disorders of Eye and Adnexa) | .0021 (.0003 - .0068) |
| Diagnosis Code: H6 (Diseases of External Ear; Diseases of Middle Ear and Mastoid) | .0033 (.0003 - .0114) |
| Diagnosis Code: H9 (Other Disorders of Ear) | .0032 (.0004 - .0104) |
| Diagnosis Code: J0 (Acute Upper Respiratory Infections) | .0059 (.0012 - .0166) |
| Diagnosis Code: J1 (Influenza and Pneumonia) | .0094 (.0015 - .0239) |
| Diagnosis Code: J2 (Other Acute Lower Respiratory Infections) | .0042 (.0008 - .0127) |
| Diagnosis Code: J3 (Other Diseases of Upper Respiratory Tract) | .0033 (.0007 - .0089) |
| Diagnosis Code: J4 (Chronic Lower Respiratory Diseases) | .0052 (.0007 - .0173) |
| Diagnosis Code: K0 (Diseases of Oral Cavity, Salivary Glands, and Jaws) | .0039 (.0004 - .0121) |
| Diagnosis Code: K2 (Diseases of Oesophagus, Stomach, and Duodenum) | .0023 (.0005 - .0067) |
| Diagnosis Code: K5 (Noninfective Enteritis and Colitis; Other Diseases of Intestines) | .0035 (.0006 - .0107) |
| Diagnosis Code: L0 (Infections of the Skin and Subcutaneous Tissue) | .0020 (.0001 - .0082) |
| Diagnosis Code: L3 (Dermatitis and Eczema) | .0017 (.0002 - .0063) |
| Diagnosis Code: M2 (Arthropathies) | .0040 (.0006 - .0104) |
| Diagnosis Code: N3 (Other Diseases of Urinary System) | .0025 (.0004 - .0069) |
| Diagnosis Code: N4 (Diseases of Male Genital Organs) | .0015 (.0001 - .0058) |
| Diagnosis Code: P0 (Fetus and Newborn Affected by Maternal Factors and by Complications of Pregnancy, Labour, and Delivery; Disorders Related to Gestation and Fetal Growth) | .0031 (.0003 - .0108) |
| Diagnosis Code: P2 (Respiratory and Cardiovascular Disorders Specific to the Perinatal Period) | .0037 (.0004 - .0110) |
| Diagnosis Code: P3 (Infections Specific to the Perinatal Period) | .0015 (.0001 - .0049) |
| Diagnosis Code: P5 (Haemorrhagic and Haematological Disorders Specific to Fetus and Newborn) | .0047 (.0004 - .0164) |
| Diagnosis Code: P7 (Transitory Endocrine and Metabolic Disorders Specific to Fetus and Newborn; Conditions Involving the Integument and Temperature Regulation of Fetus and Newborn) | .0016 (.0001 - .0063) |
| Diagnosis Code: P9 (Other Disorders Originating in the Perinatal Period) | .0026 (.0003 - .0104) |
| Diagnosis Code: Q2 (Congenital Malformations of the Circulatory System) | .0054 (.0006 - .0154) |
| Diagnosis Code: Q5 (Congenital Malformations of Genital Organs) | .0014 (.0002 - .0045) |
| Diagnosis Code: Q6 (Congenital Malformations of the Urinary System; Congenital Malformations and Deformations of the Musculoskeletal System) | .0033 (.0004 - .0108) |
| Diagnosis Code: R0 (Symptoms and Signs Involving the Circulatory and Respiratory Systems) | .0061 (.0001 - .0197) |
| Diagnosis Code: R1 (Symptoms and Signs Involving the Digestive System and Abdomen) | .0032 (.0004 - .0090) |
| Diagnosis Code: R2 (Symptoms and Signs Involving the Nervous and Musculoskeletal Systems) | .0045 (.0007 - .0156) |
| Diagnosis Code: R4 (Symptoms and Signs Involving Cognition, Perception, Emotional State and Behaviour) | .0607 (.0464 - .0761) |
| Diagnosis Code: R5 (General Symptoms and Signs) | .0042 (.0009 - .0119) |
| Diagnosis Code: R6 (General Symptoms and Signs) | .0036 (.0006 - .0105) |
| Diagnosis Code: S0 (Injuries to the Head) | .0038 (.0008 - .0109) |
| Diagnosis Code: S5 (Injuries to the Elbow and Forearm) | .0084 (.0018 - .0109) |
| Diagnosis Code: S6 (Injuries to the Wrist and Hand) | .0053 (.0007 - .0137) |
| Diagnosis Code: S8 (Injuries to the Knee and Lower Leg) | .0014 (.0002 - .0052) |
| Diagnosis Code: T1 (Injuries to Unspecified Part of Trunk, Limb, or Body Region; Effects of Foreign Body Entering Through Natural Orifice) | .0018 (.0002 - .0066) |
| Diagnosis Code: T3 (Burns and Corrosions; Frostbite; Poisoning by Drugs, Medicaments and Biological Substances) | .0504 (.0382 - .0624) |
| Diagnosis Code: T4 (Poisoning by Drugs, Medicaments and Biological Substances) | .0278 (.0185 - .0392) |
| Diagnosis Code: T8 (Complications of Surgical and Medical Care, Not Elsewhere Classified) | .0026 (.0004 - .0102) |
| Diagnosis Code: U5 (Provisional Assignment of New Diseases of Uncertain Etiology or Emergency Use) | .0151 (.0042 - .0283) |
| Diagnosis Code: W0 (Falls) | .0042 (.0006 - .0130) |
| Diagnosis Code: W1 (Falls) | .0031 (.0007 - .0087) |
| Diagnosis Code: W2 (Exposure to Inanimate Mechanical Forces) | .0021 (.0003 - .0070) |
| Diagnosis Code: X4 (Accidental Poisoning by and Exposure to Noxious Substances) | .0043 (.0007 - .0122) |
| Diagnosis Code: X5 (Overexertion, Travel and Privation; Accidental Exposure to Other and Unspecified Factors) | .0017 (.0002 - .0063) |
| Diagnosis Code: Z0 (Persons Encountering Health Services for Examination and Investigation) | .0136 (.0020 - .0286) |
| Diagnosis Code: Z3 (Persons Encountering Health Services in Circumstances Related to Reproduction) | .0053 (.0013 - .0145) |
| Diagnosis Code: Z4 (Persons Encountering Health Services for Specific Procedures and Health Care) | .0019 (.0001 - .0058) |
| Diagnosis Code: Z5 (Persons Encountering Health Services for Specific Procedures and Health Care; Persons with Potential Health Hazards Related to Socioeconomic and Psychosocial Circumstances) | .0030 (.0006 - .0091) |
| Diagnosis Code: Z6 (Persons with Potential Health Hazards Related to Socioeconomic and Psychosocial Circumstances) | .0163 (.0054 - .0277) |
| Diagnosis Code: Z7 (Persons Encountering Health Services in Other Circumstances) | .0047 (.0007 - .0120) |
| Diagnosis Code: Z8 (Persons with Potential Health Hazards Related to Family and Personal History and Certain Conditions Influencing Health Status) | .0048 (.0008 - .0171) |
| Diagnosis Code: Z9 (Persons with Potential Health Hazards Related to Family and Personal History and Certain Conditions Influencing Health Status) | .0542 (.0380 - .0709) |
| Disability (Memory): Has Disability | .0272 (.0100 - .0492) |
| Disability (Mobility): Has Disability | .0152 (.0036 - .0326) |
| Disability (None): Has Disability | .0507 (.0199 - .0838) |
| Disability (Sensory): Has Disability | .0377 (.0174 - .0613) |
| Ethnicity: Asian | .0139 (.0050 - .0239) |
| Ethnicity: Black, African, Caribbean or Black British | .0038 (.0005 - .0106) |
| Ethnicity: Mixed Ethnic Groups | .0023 (.0003 - .0076) |
| Ethnicity: Information not Obtained | .0025 (.0004 - .0083) |
| Ethnicity: Other Ethnic Group | .0035 (.0004 - .0099) |
| Ethnicity: Information Refused | .0015 (.0000 - .0052) |
| School Exclusion Category: Fixed Term Exclusion | .1810 (.1514 - .2092) |
| School Exclusion Category: Permanent Exclusion | .0060 (.0013 - .0122) |
| Free School Meal Status: Eligible | .0229 (.0050 - .0514) |
| Free School Meal Status: Unknown | .0003 (.0000 - .0011) |
| Gender: Female | .0756 (.0434 - .1056) |
| Gestation Age: Continuous Value | .0169 (.0086 - .0329) |
| Autistic Spectrum Disorder Status: Autistic | .1124 (.0893 - .1377) |
| Autistic Spectrum Disorder Status: Unknown | .0000 (.0000 - .0000) |
| Dental Check Status: Not Receiving Checks | .0984 (.0656 - .1354) |
| Dental Check Status: Unknown | .0067 (.0020 - .0191) |
| Immunisation Status: Not Immunised | .0711 (.0408 - .1071) |
| Immunisation Status: Unknown | .0032 (.0008 - .0091) |
| Substance Misuse: Misusing Substances | .1933 (.1750 - .2127) |
| Substance Misuse: Unknown | .0002 (.0000 - .0006) |
| Labour Onset: Caesarean Section | .0030 (.0004 - .0085) |
| Labour Onset: Surgical Induction (amniotomy) | .0093 (.0013 - .0194) |
| Labour Onset: Medical Induction | .0008 (.0000 - .0031) |
| Labour Onset: Onset Not Known | .0007 (.0000 - .0032) |
| Labour Onset: Unknown | .0405 (.0119 - .0752) |
| Looked After Child Status: Looked After | .2187 (.1878 - .2482) |
| Maternal Smoking: Gave up during pregnancy | .0007 (.0000 - .0027) |
| Maternal Smoking: 0-9 cigarettes per day | .0024 (.0004 - .0080) |
| Maternal Smoking: 10-19 cigarettes per day | .0028 (.0004 - .0075) |
| Maternal Smoking: 20-29 cigarettes per day | .0004 (.0000 - .0018) |
| Maternal Smoking: Non-smoker | .0023 (.0004 - .0073) |
| Maternal Smoking: Unknown | .0122 (.0022 - .0309) |
| Apgar 1-Minute Score: Unknown | .0166 (.0025 - .0372) |
| Apgar 5-Minute Score: Unknown | .0400 (.0178 - .0686) |
| Birth Weight: Unknown | .0045 (.0006 - .0148) |
| Gestation Age: Unknown | .0042 (.0006 - .0125) |
| Welsh Index of Multiple Deprivation: Unknown | .0003 (.0000 - .0014) |
| Category of Need: Abuse or Neglect | .0647 (.0327 - .0966) |
| Category of Need: Child’s Disability or Illness | .0273 (.0068 - .0547) |
| Category of Need: Parental Disability or Illness | .0023 (.0003 - .0079) |
| Category of Need: Family in Acute Stress | .0605 (.0348 - .0860) |
| Category of Need: Family Dysfunction | .0048 (.0014 - .0117) |
| Category of Need: Socially Unacceptable Behaviour | .0200 (.0108 - .0318) |
| Category of Need: Low Income | .0004 (.0000 - .0019) |
| Category of Need: Absent Parenting | .0023 (.0003 - .0076) |
| Category of Need: Adoption Disruption | .0013 (.0000 - .0038) |
| Operation Code: A5 (Other operations on meninges of spinal cord; Therapeutic epidural injection; Drainage of spinal canal; Therapeutic spinal puncture; Diagnostic spinal puncture; Operations on spinal nerve root; Excision of peripheral nerve) | .0016 (.0001 - .0057) |
| Operation Code: D1 (Exenteration of mastoid air cells; Other operations on mastoid; Attachment of bone anchored hearing prosthesis; Repair of eardrum; Drainage of middle ear; Reconstruction of ossicular chain; Other operations on ossicle of ear; Extirpation of lesion of middle ear) | .0018 (.0003 - .0055) |
| Operation Code: E2 (Operations on adenoid; Repair of pharynx; Other open operations on pharynx; Therapeutic endoscopic operations on pharynx; Diagnostic endoscopic examination of pharynx; Other operations on pharynx; Operations on cricopharyngeus muscle; Excision of larynx) | .0029 (.0003 - .0100) |
| Operation Code: F1 (Simple extraction of tooth; Preprosthetic oral surgery; Surgery on apex of tooth; Restoration of tooth; Orthodontic operations; Other orthodontic operations; Other operations on tooth; Operations on teeth using dental crown or bridge; Excision of dental lesion of jaw) | .0053 (.0007 - .0171) |
| Operation Code: F3 (Other repair of palate; Other operations on palate; Excision of tonsil; Other operations on tonsil; Extirpation of lesion of other part of mouth; Reconstruction of other part of mouth) | .0036 (.0009 - .0085) |
| Operation Code: G4 (Incision of pylorus; Other operations on pylorus; Other fibreoptic endoscopic extirpation of lesion of upper gastrointestinal tract; Fibreoptic endoscopic extirpation of lesion of upper gastrointestinal tract; Other therapeutic fibreoptic endoscopic operations on upper gastrointestinal tract; Diagnostic fibreoptic endoscopic examination of upper gastrointestinal tract; Therapeutic fibreoptic endoscopic operations on upper gastrointestinal tract; Intubation of stomach; Other operations on stomach; Excision of duodenum.) | .0018 (.0001 - .0061) |
| Operation Code: S4 (Other closure of skin; Suture of skin of head or neck; Suture of skin of other site; Removal of repair material from skin; Removal of other inorganic substance from skin; Removal of other substance from skin; Opening of skin; Insertion of skin expander into subcutaneous tissue; Attention to skin expander in subcutaneous tissue) | .0037 (.0006 - .0106) |
| Operation Code: S5 (Introduction of other inert substance into subcutaneous tissue; Introduction of destructive substance into subcutaneous tissue; Introduction of therapeutic substance into subcutaneous tissue, Introduction of substance into skin; Exploration of burnt skin of head or neck; Exploration of burnt skin of other site; Exploration of other skin of head or neck; Exploration of other skin of other site; Larvae therapy of skin; Leech therapy of skin) | .0023 (.0005 - .0059) |
| Operation Code: U0 (Diagnostic imaging of whole body; Diagnostic imaging of mouth; Diagnostic imaging of central nervous system; Diagnostic imaging of face and neck; Diagnostic imaging of chest; Diagnostic imaging of abdomen; Diagnostic imaging of pelvis) | .0196 (.0049 - .0343) |
| Operation Code: U2 (Diagnostic echocardiography; Diagnostic imaging procedures; Neuropsychology tests; Nuclear medicine haematological tests, Diagnostic audiology, Breath tests, Diagnostic testing of genitourinary system; Diagnostic application tests on skin; Other diagnostic tests on skin, Diagnostic endocrinology) | .0039 (.0006 - .0131) |
| Operation Code: W2 (Primary open reduction of fracture of bone and extramedullary fixation; Primary open reduction of intra-articular fracture of bone; Other primary open reduction of fracture of bone; Secondary open reduction of fracture of bone; Closed reduction of fracture of bone and internal fixation; Closed reduction of fracture of bone and external fixation; Other closed reduction of fracture of bone; Fixation of epiphysis; Other internal fixation of bone; Skeletal traction of bone) | .0027 (.0006 - .0064) |
| Operation Code: X2 (Correction of congenital deformity of forearm; Correction of congenital deformity of hand; Correction of congenital deformity of hip; Correction of congenital deformity of leg; Primary correction of congenital deformity of foot; Other correction of congenital deformity of foot; Correction of minor congenital deformity of foot; Intermittent infusion of therapeutic substance; Continuous Infusion of therapeutic substance) | .0021 (.0003 - .0059) |
| Operation Code: X3 (Injection of therapeutic substance; Injection of radiocontrast material; Exchange blood transfusion; Other blood transfusion; Other intravenous transfusion; Other intravenous injection; Blood withdrawal; Intramuscular injection; Subcutaneous injection; Other route of administration of therapeutic substance) | .0026 (.0002 - .0093) |
| Operation Code: X5 (External resuscitation; Change of body temperature; Oxygen therapy; Extirpation of unspecified organ; Other operations on unspecified organ; Intubation of trachea; Artificial support for body system; Anaesthetic without surgery) | .0018 (.0002 - .0093) |
| Operation Code: Y5 (Approach through abdominal cavity; Approach to organ through artificial opening into gastrointestinal tract; Approach to organ through other opening; Approach to organ under image control; Harvest of nerve; Harvest of random pattern flap of skin from limb; Harvest of random pattern flap of skin from other site; Harvest of axial pattern flap of skin; Harvest of skin for graft; Harvest of flap of skin and fascia) | .0061 (.0009 - .0165) |
| Operation Code: Y7 (Early operations NOC; Late operations NOC; Facilitating operations NOC; Minimal access to thoracic cavity; Minimal access to abdominal cavity; Minimal access to other body cavity; Arteriotomy approach to organ under image control; Approach to organ through artery) | .0013 (.0002 - .0040) |
| Operation Code: Y8 (General Anaesthetic; Spinal Anaesthetic; Local Anaesthetic; Other Anaesthetic; Y89 Brachytherapy) | .0061 (.0019 - .0153) |
| Operation Code: Y9 (Other non-operations; External beam radiotherapy; Support for preparation for radiotherapy; Gallium-67 imaging; Radiopharmaceutical imaging; Gestational age; In vitro fertilisation; Radiology with contrast; Y98 Radiology procedures; Y99 Donor status) | .0094 (.0017 - .0232) |
| Operation Code: Z4 (Other vascular tissue; Upper urinary tract; Lower urinary tract; Male genital organ; Vagina; Uterus; Other female genital tract; Skin of face; Skin of other part of head or neck; Skin of trunk) | .0018 (.0002 - .0056) |
| Operation Code: Z5 (Skin of other site; Nail; Chest wall; Abdominal wall; Muscle of shoulder or upper arm; Muscle of forearm; Muscle of hand; Muscle of hip or thigh; Muscle of lower leg; Muscle of foot) | .0032 (.0003 - .0106) |
| Operation Code: Z7 (Radius; Ulna; Other bone of arm or wrist; Other bone of hand; Rib cage; Bone of pelvis; Femur; Tibia; Bone of tarsus) | .0040 (.0003 - .0130) |
| Operation Code: Z8 (Other bone of foot; Joint of shoulder girdle or arm; Joint of wrist or hand; Joint of finger; Joint of pelvis or upper leg; Joint of lower leg or tarsus; Other joint of foot; Other part of musculoskeletal system; Respiratory tract; Arm region) | .0017 (.0003 - .0050) |
| Operation Code: Z9 (Leg region; Other vein of upper body; Other region of body; Other veins of pelvis; Laterality of operation; Other branch of thoracic aorta; Other lateral branch of abdominal aorta; Other terminal branch of aorta; Other veins of lower limb; Intervertebral disc) | .0076 (.0016 - .0225) |
| Parenting Capacity (Domestic Abuse): Abuse | .0551 (.0253 - .0890) |
| Parenting Capacity (Domestic Abuse): Unknown | .0006 (.0000 - .0016) |
| Parenting Capacity (Learning Disabilities): Learning Disabilities | .0043 (.0006 - .0147) |
| Parenting Capacity (Learning Disabilities): Unknown | .0006 (.0000 - .0015) |
| Parenting Capacity (Mental Health): Mental Health Issues | .1395 (.1023 - .1764) |
| Parenting Capacity (Mental Health): Unknown | .0012 (.0000 - .0043) |
| Parenting Capacity (Physical Health): Physical Health Issues | .0109 (.0020 - .0310) |
| Parenting Capacity (Physical Health): Unknown | .0008 (.0001 - .0021) |
| Parenting Capacity (Substance Misuse): Substance Misuse | .0667 (.0352 - .1020) |
| Parenting Capacity (Substance Misuse): Unknown | .0010 (.0000 - .0032) |
| Welsh Index of Multiple Deprivation: Continuous Value | .0800 (.0512 - .1112) |
| Youth Offending Status: Offender | .0305 (.0154 - .0470) |
| Youth Offending Status: Unknown | .0008 (.0000 - .0023) |

**Table 7a: Fairness Metrics (Gradient Boosting Classifier)**

|  | **TPR** | **TNR** | **PPV** | **NPV** |
| --- | --- | --- | --- | --- |
| **Asian** | 0.091 | 1.0 | 1.0 | 0.861 |
| **Black** | 0.0 | 1.0 | 0.0 | 0.918 |
| **Mixed** | 0.154 | 0.935 | 0.286 | 0.867 |
| **Other Ethnicity** | 0.400 | 1.0 | 1.0 | 0.938 |
| **White** | 0.166 | 0.979 | 0.657 | 0.825 |
| **Female** | 0.086 | 0.984 | 0.525 | 0.839 |
| **Male** | 0.232 | 0.972 | 0.701 | 0.818 |

*TPR: True Positive Rate (Sensitivity)*

*TNR: True Negative Rate (Specificity)*

*PPV: Positive Predictive Value*

*NPV: Negative Predictive Value*

**Table 7b: Fairness Metrics (Logistic Regression Model)**

|  | **TPR** | **TNR** | **PPV** | **NPV** |
| --- | --- | --- | --- | --- |
| **Asian** | 0.364 | 0.935 | 0.5 | 0.892 |
| **Black** | 0.0 | 0.933 | 0.0 | 0.913 |
| **Mixed** | 0.538 | 0.688 | 0.226 | 0.898 |
| **Other Ethnicity** | 1.0 | 0.844 | 0.417 | 1.0 |
| **White** | 0.592 | 0.700 | 0.330 | 0.873 |
| **Female** | 0.560 | 0.731 | 0.301 | 0.889 |
| **Male** | 0.599 | 0.689 | 0.352 | 0.859 |

*TPR: True Positive Rate (Sensitivity)*

*TNR: True Negative Rate (Specificity)*

*PPV: Positive Predictive Value*

*NPV: Negative Predictive Value*
